# Corneal stromal microdots accumulation and its association with corneal neurodegeneration and retinal microvascular perfusion in diabetes

**DOI:** 10.3389/fendo.2025.1481018

**Published:** 2025-08-27

**Authors:** Jingrao Wang, Shu Wang, Hong Zhang, Xin Jin

**Affiliations:** Eye Hospital, The First Affiliated Hospital of Harbin Medical University, Key Laboratory of Basic and Clinical Research of Heilongjiang Province, Harbin, Heilongjiang, China

**Keywords:** microdots, lipofuscin, *in vivo* confocal microscopy (IVCM), optical coherence tomography angiography (OCTA), diabetes mellitus (DM)

## Abstract

**Purpose:**

The purpose of the study was to investigate the deposition of microdots in corneal stroma in diabetes mellitus (DM) patients and assess the relationship between microdots and corneal nerve damage as well as retinal microvascular perfusion.

**Methods:**

127 patients with DM and 36 age and sex-matched controls were included. All subjects were examined by *in vivo* confocal microscopy (IVCM) and optical coherence tomography angiography (OCTA). Histochemistry staining was also performed in cadaver corneas from patients with DM or control subjects.

**Results:**

The deposition of microdots was significantly increased in corneal stroma in patients with DM than controls (*P*<0.0001). Spearman’s rank correlation showed that the deposition of microdots in subepithelial, anterior stromal, mid stromal, posterior stromal, and pre-Descemet stromal layers had positive correlations with corneal nerve fiber density (CNFD) (*q*<0.0001, *q*<0.0001, *q*<0.0001, *q*=0.0039, and *q*=0.0104), corneal nerve fiber length (CNFL) (*q*<0.0001, *q*<0.0001, *q*<0.0001, *q*<0.0001, and *q*<0.0001), and corneal tortuosity (*q*<0.0001, *q*<0.0001, *q*<0.0001, *q*<0.0001, and *q*<0.0001). The subepithelial, anterior stromal, and mid stromal microdots also showed weak correlations with superficial vascular complexes (SVC) vessel density (VD) (*q*<0.0001, *q*=0.0011, and *q*=0.0004) and deep vascular complexes (DVC) VD (*q=*0.0001, *q*=0.0109, and *q*=0.0037). PAS and long Ziehl-Neelsen staining demonstrated lipofuscin deposits in cornea.

**Conclusion:**

Patients with DM demonstrated a significant increase of deposition of microdots in corneal stroma which correlates with corneal nerve loss and retinal microvascular perfusion. Microdots are at least partially composed of lipofuscin which is also observed in corneal basal epithelial layer.

## 1 Introduction

Diabetes mellitus (DM) is a metabolic disorder of complex etiology characterized by chronic hyperglycemia with disturbed metabolism of carbohydrate, fat, and protein resulting from defects in insulin secretion, insulin action, or both (1). Sustained hyperglycemia eventually results in ocular complications including diabetic keratopathy (DK) and diabetic retinopathy (DR) which may lead to vision loss (2). The hyperglycemic environment of the DM cornea can affect epithelial layer and endothelial layer causing persistent corneal epithelial defects and increased corneal thickness (3). Also, DM can affect stromal layer causing increased stiffness (4). The above changes make DK a complicated clinical condition that is challenging to treat.

*In vivo* confocal microscopy (IVCM) is a noninvasive examination with histologic resolution that allows for and can be used to observe the subtle changes in the stroma. Microdots were first found as a type of chronic stromal change in patients with long-term contact lens wear (5). Later, microdots have also been reported in patients with Thygeson’s superficial punctate keratopathy (6), Reis-Bücklers’ corneal dystrophy (7), Fabry disease (8, 9), and patients after corneal surgery (10). However, there’s no study focused on microdots in DM corneas. As highly reflective with the size of 0.3-2μm, microdots were found not only in patients with corneal disease but also in healthy corneas and were increased with age (11, 12). These microdots were hypothesized as lipofuscin-like material that accumulates as a result of chronic oxygen deprivation and chronic microtrauma to the cornea (5, 13). Priyadarsini et al. (14) have found altered lipid retention, which was an important component of lipofuscin, in the corneal stroma using untargeted mass spectrometry. So, we hypothesized that the deposition of microdots might change in DM corneal stroma.

The purpose of this study was to investigate the changes in corneal stromal microdots using IVCM. The further aim was to determine the correlation between the microdots and clinical signs of DM, corneal nerve fiber, corneal cell density in variable layers, and retinal microvascular changes.

## 2 Materials and methods

### 2.1 Participants

This is a single center study that evaluated 163 participants, including 127 with diabetes and 36 healthy control subjects between 2018 and 2023. We included adult patients with diabetes. Exclusion criteria were non-diabetic systemic diseases affecting the cornea, cornea disorders, active intraocular inflammation, prior corneal surgery, history of contact lens wear, use of ocular medication. All subjects underwent comprehensive eye examinations including slit-lamp examination, intraocular pressure, visual acuity, IVCM, OCTA, fundus examination. Venous blood sampling was performed for glycated hemoglobin level (HbA1c) and lipid profile tests.

### 2.2 OCTA

All images were obtained by AngioVue^®^ OCT angiography (OCTA) (Optovue, Inc., Fremont, CA, USA). For each scan, superficial and deep OCTA images were generated based on fully automated retinal segmentation performed by the OCTA device software. The top and bottom layers of superficial vascular complexes (SVC) were defined as the inner limiting membrane (ILM) and the inner plexiform layer (IPL) with an offset of 10μm, respectively. The top and bottom layers of the deep vascular complexes (DVC) were defined as the IPL with an offset of 10μm and the underlying outer plexiform layer plus Henle’s fiber layer (OPL) with an offset of 10μm. VD was defined as the proportion of blood flow signal detected by OCTA to the corresponding area. The FAZ area was defined as the region surrounding the fovea devoid of any retinal capillaries on SVP images. The numerical value was calculated automatically by the OCTA software.

### 2.3 *In vivo* confocal microscopy image acquisition and analysis

Laser scanning IVCM (Heidelberg Retina Tomograph 3 with Rostock Cornea Module, Heidelberg Engineering GmbH, Heidelberg, Germany) images of the central cornea were obtained from all subjects. Before the examination, one drop of 0.4% oxybuprocaine hydrochloride (Benoxil; Santen Pharmaceutical, Japan) was applied to the lower conjunctival sac. Three representative images of each structure in each eye and each layer were selected for analysis.

Microdots were defined as 1-2μm highly reflective round structures within each 400 × 400μm images, and were manually measured using the Heidelberg cell counting software and graded as follows: Grade 0 (<5 dots), Grade 1 (5–10 dots), Grade 2 (11–25 dots), Grade 3 (26–50 dots), Grade 4 (51–75 dots), and Grade 5 (>75 dots) (11). Five stromal layer were defined as subepithelial layer (0-10% of the stromal depth), anterior stromal layer (11-30% of the stromal depth), mid stromal layer (31-60% of the stromal depth), posterior stromal layer (61-90% of the stromal depth), and pre-Descemet stromal layer (91-100% of the stromal depth) (11).

The density (cells/mm^2^) of basal epithelial cells, endothelial cells, and inflammatory cells was manually quantified using the Heidelberg cell counting software.

The following parameters of the corneal sub-basal nerve plexus were recorded: corneal nerve fiber density (CNFD), the number of major nerve fibers per mm^2^; corneal nerve branch density (CNBD), the number of branch points on the main fibers per mm^2^; corneal nerve fiber length (CNFL), the total length of nerves in mm per mm^2^; and tortuosity, the morphology of the nerves. CNFD, CNBD, CNFL were counted manually per frame using Image J (National Institutes of Health, USA) and Neuron J (https://www.imagescience.org/meijering/software/neuronj/), a semiautomated nerve analysis plug-in program for Image J. Tortuosity was qualitatively classified into 5 grades according to the Oliveira-Soto and Efron scale (15).

### 2.4 Histochemistry staining

Fixed cornea tissue was sectioned, and mounted on slides. For Hematoxylin-Eosin (HE) staining, the sections were incubated in Hematoxylin solution for 15min, then differentiation solution for 3min, and Eosin Y aqueous solution for 1min. For periodic acid-Schiff (PAS) staining, tissues were oxidized in 0.5% periodic acid solution for 5 min, Schiff’s reagent for 15min and, Mayer’s hematoxylin for 1min using PAS Stain Kit (Solarbio). For long Ziehl-Neelsen staining, a commercially available lipofuscin staining detection kit (Solarbio) was used in accordance with the manufacturer’s instructions. The sections were incubated in the fuchsin solution overnight, followed with long acid differentiation solution until the background staining was removed. Then, the sections were incubated in methylene blue solution for 1min. After staining, the sections were dehydrated and mounted. Finally, stained sections were imaged using an microscope (Olympus).

### 2.5 Statistical analysis

Data were analyzed using the SPSS software version 22.0 (SPSS Inc., IBM, Chicago, IL, USA) and GraphPad Prism 9 for Windows (version 9.0.0; GraphPad Software). Data normality was tested with the Kolmogorov-Smirnov test. The χ^2^ test was used to analyze sex differences. The t-test or Mann-Whitney U test was used to compare other variables between the two groups. One-way analysis of variance or the Kruskal-Wallis H test was used to compare data among multiple groups. Spearman correlation coefficients with FDR correction quantified the association between microdots and other variables. The level of statistical significance was set at p<0.05 and was adjusted by Bonferroni correction when pairwise comparison in multiple groups was conducted.

## 3 Results

### 3.1 Demographics

This study evaluated 127 eyes from 127 patients with DM and 36 eyes from 36 healthy volunteers. The demographic data of the patients and control subjects are presented in **Table 1**. No significant differences in age or sex were seen between the DM and control groups. Among patients with DM, 75 eyes without DR, 33 eyes had NPDR, and 19 eyes had PDR.

**Table 1:** Demographics and clinical data for patients with DM and the control subjects.

Variable	Control subjects (n = 36)	Diabetes (n =127)	χ2/Z Value	*P* Value
Age (years)	58.5 (42.5, 65)	57 (49, 65)	-0.51	0.61
Sex, male/female	19/17	71/56	0.11	0.74
Duration of diabetes (years)	–	10 (4, 16)	–	–
HbA1c (%)	–	8.35 (7.10-10.08)	–	–
Total cholesterol (mmol/L)	–	4.97 ± 1.13	–	–
Triglycerides (mmol/L)	–	1.95 (1.42, 2.53)	–	–
HDL cholesterol (mmol/L)	–	1.10 ± 0.22	–	–
LDL cholesterol (mmol/L)	–	3.28 ± 0.85	–	–
VLDL cholesterol (mmol/L)	–	0.40 (0.31, 0.53)	–	–
ApoA (g/L)	–	1.33 ± 0.23	–	–
ApoB (g/L)	-	1.03 ± 0.26	-	-

Data are shown as mean ± standard deviation or median (Q25-Q75). HDL, high density lipoprotein; LDL, low density lipoprotein; VLDL, very low density lipoprotein; ApoA, Apolipoprotein A; ApoB, Apolipoprotein B.

### 3.2 Changes of corneal stromal microdots in patients with DM

**Figure 1:**
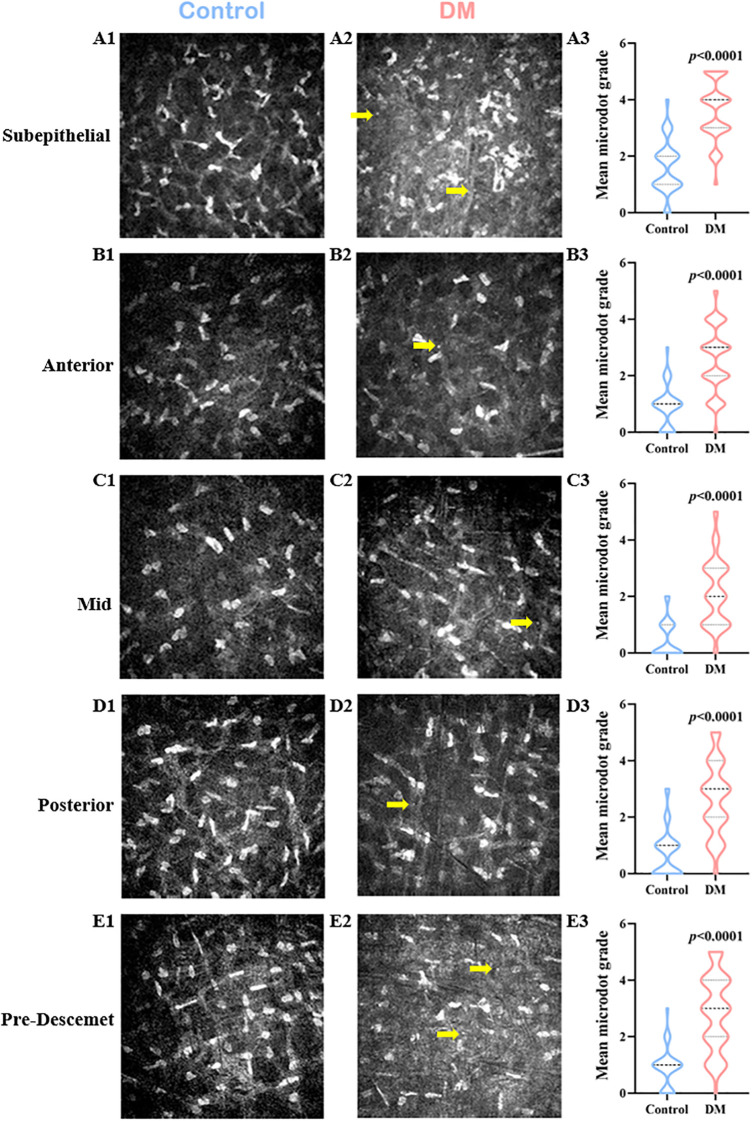
Changes of Corneal Stromal Microdots in DM patients. *In vivo* confocal microscopy (IVCM) images of the control group **(A1-E1)**, the DM group **(A2-E2)**, and comparison of the microdot (yellow arrow) grade between the two groups **(A3-E3)** in the subepithelial layer **(A1-A3)**, anterior stromal layer **(B1-B3)**, mid stromal layer **(C1-C3)**, posterior stromal layer **(D1-D3)**, and pre-Descemet stromal layer **(E1-E3)** of corneal stroma.

The deposition of microdots in the stroma is shown in **Figures 1A1-E2**. Compared with control volunteers, patients with DM had a significantly higher microdots grade in each layer of corneal stroma including the subepithelial layer (4 [Q25-Q75: 3-4] vs. 1.5 [Q25-Q75: 1-2], respectively, *P*<0.0001), anterior stromal layer (3 [Q25-Q75: 2-3] vs. 1 [Q25-Q75: 0-1], respectively, *P*<0.0001), mid stromal layer (2 [Q25-Q75: 1-3] vs. 0 [Q25-Q75: 0-1], respectively, *P*<0.0001), posterior stromal layer (3 [Q25-Q75: 2-4] vs. 1 [Q25-Q75: 0-1], respectively, *P*<0.0001), and pre-Descemet stromal layer (3 [Q25-Q75: 2-4] vs. 1 [Q25-Q75: 0-1], respectively, *P*<0.0001). The microdots most concentrated in the subepithelial layer in DM patients (**Supplementary Figure 1**).

### 3.3 Correlation of corneal stromal microdots with clinical variables in patients with DM

Subepithelial microdots showed a low positive correlation with age (r_s_=0.378; *q*=0.0001) and the duration of DM (r_s_=0.286; q=0.0052). Anterior stromal microdots (r_s_=0.326; *q*=0.0009) and mid stromal microdots (r_s_=0.326; *q*=0.0010) also showed a low positive correlation with age. There was no correlation between microdot in other stromal layers and clinical variables (**Table 2**).

**Table 2:** Correlation of corneal stromal microdots and clinical variables.

Grade of microdots	Subepithelial layer (n=127)		Anterior stromal layer (n=127)		Mid stromal layer (n=127)		Posterior stromal layer (n=127)		Pre-Descemet stromal layer (n=127)	
	r_s_	*q*	r_s_	*q*	r_s_	*q*	r_s_	*q*	r_s_	*q*
Age	**0.378**	**0.0001**	**0.326**	**0.0009**	**0.326**	**0.0010**	**0.253**	**0.0133**	0.176	0.11
Gender	-0.198	0.07	-0.145	0.22	-0.117	0.33	-0.126	0.29	-0.095	0.45
Duration of DM	**0.286**	**0.0052**	0.170	0.13	0.196	0.08	0.111	0.36	0.057	0.72
HbA1c	-0.058	0.76	-0.027	0.87	0.031	0.86	-0.039	0.84	-0.078	0.64
TC	0.188	0.15	0.210	0.09	0.120	0.38	0.138	0.32	0.106	0.47
TG	0.051	0.78	-0.038	0.84	0.013	0.90	-0.025	0.87	0.030	0.85
HDL	0.041	0.84	-0.015	0.91	-0.024	0.89	0.073	0.73	0.091	0.63
LDL	0.261	0.08	0.214	0.17	0.142	0.39	0.166	0.32	0.105	0.58
VLDL	-0.047	0.84	-0.037	0.87	-0.048	0.83	-0.045	0.83	-0.062	0.78
ApoA	0.023	0.90	-0.117	0.51	-0.093	0.63	-0.053	0.82	0.018	0.91
ApoB	0.244	0.10	0.272	0.07	0.187	0.25	0.203	0.20	0.163	0.32

Correlations between various analyzed parameters were calculated using Spearman rank test. TC, total cholesterol; TG, triglycerides. FDR correction was applied for multiple correlation analysis.

Results with statistical significance are indicated in bold.

### 3.4 Changes of corneal nerve and corneal cell density in patients with DM

In comparison with control subjects, patients with DM had lower basal epithelial cell density (6144.5 [Q25-Q75: 5853.5-6417.5] vs. 5876 [Q25-Q75:5213-6363], respectively, *P*=0.0210), CNFD (32 [Q25-Q75: 26-39] vs. 17 [Q25-Q75: 13-25], respectively, *P*<0.0001), CNBD (80 [Q25-Q75: 59.5-99.75] vs. 57 [Q25-Q75: 44-71], respectively, *P*<0.0001), CNFL (18.65 ± 3.55 vs. 11.38 ± 4.38, respectively, *P*<0.0001), and corneal endothelial cell density (3104[Q25-Q75: 2726.75-3295] vs. 2778 [Q25-Q75: 2479-3024], respectively, *P*=0.0003). Meanwhile, the density of DCs (4 [Q25-Q75: 0-13] vs. 13 [Q25-Q75: 6-27], respectively, *P*<0.0001) and the grade of tortuosity (1 [Q25-Q75: 1-1] vs. 2 [Q25-Q75: 2-3], respectively, *P*<0.0001) were higher in patients with DM than in control subjects (**Supplementary Table 1**).

### 3.5 Correlation of microdots with corneal nerve and corneal cell density in patients with DM

Spearman’s rank correlation showed that the deposition of microdots in stroma had a negative correlation with CNFD (subepithelial layer, r_s_=-0.451, *q*<0.0001; anterior stromal layer, r_s_=-0.388, *q*<0.0001; mid stromal layer, r_s_=-0.402, *q*<0.0001; posterior stromal layer, r_s_=-0.290, *q*=0.0039; and pre-Descemet layer, r_s_=-0.264, *q*=0.0104), CNBD (subepithelial layer, r_s_=-0.362, *q*=0.0002; anterior stromal layer, r_s_=-0.258, *q*=0.0118; mid stromal layer, r_s_=-0.210, *q*=0.05; posterior stromal layer, r_s_=-0.186, *q*=0.09; and pre-Descemet layer, r_s_=-0.215, *q*=0.0458), and CNFL (subepithelial layer, r_s_=-0.684, *q*<0.0001; anterior stromal layer, r_s_=-0.564, *q*<0.0001; mid stromal layer, r_s_=-0.546, *q*<0.0001; posterior stromal layer, r_s_=-0.526, *q*<0.0001; and pre-Descemet layer, r_s_=-0.485, *q*<0.0001). Meanwhile, the deposition of microdots had a positive correlation with corneal nerve tortuosity (subepithelial layer, r_s_=0.624, *q*<0.0001; anterior stromal layer, r_s_=0.488, *q*<0.0001; mid stromal layer, r_s_=0.469, *q*<0.0001; posterior stromal layer, r_s_=0.395, *q*<0.0001; and pre-Descemet layer, r_s_=0.409, *q*<0.0001), and DC density (subepithelial layer, r_s_=0.262, *q*=0.0106; anterior stromal layer, r_s_=0.215, *q*=0.0447; mid stromal layer, r_s_=0.217, *q*=0.0453; posterior stromal layer, r_s_=0.220, *q*=0.0419). However, the deposition of microdots in the pre-Descemet layer didn’t correlate with DC density (**Supplementary Figure 2**). Also, stromal microdots had no correlation with corneal basal epithelial cell density and endothelial cell density (**Table 3**).

**Table 3:** Correlation of corneal stromal microdots with IVCM parameters and retinal vessel density.

Grade of microdots	Basal epithelial cell density	CNFD	CNBD	CNFL	Tortuosity	DC density	Corneal endothelial density	SVC VD	DVC VD
Subepithelial layer (n=127)									
r_s_	0.01	**-0.451**	**-0.362**	**-0.684**	**0.624**	**0.262**	0.08	**-0.432**	**-0.366**
q	0.90	**<0.0001**	**0.0002**	**<0.0001**	**<0.0001**	**0.0106**	0.56	**<0.0001**	**0.0001**
Anterior stromal layer (n=127)									
r_s_	-0.05	**-0.388**	**-0.258**	**-0.564**	**0.488**	**0.215**	0.12	**-0.320**	**-0.262**
q	0.77	**<0.0001**	**0.0118**	**<0.0001**	**<0.0001**	**0.0447**	0.31	**0.0011**	**0.0109**
Mid stromal layer (n=127)									
r_s_	-0.07	**-0.402**	-0.21	**-0.546**	**0.469**	**0.217**	0.05	**-0.347**	**-0.293**
q	0.61	**<0.0001**	0.05	**<0.0001**	**<0.0001**	**0.0453**	0.75	**0.0004**	**0.0037**
Posterior stromal layer (n=127)									
r_s_	0.02	**-0.29**	-0.186	**-0.526**	**0.395**	**0.22**	0.13	**-0.276**	-0.187
q	0.90	**0.0039**	0.09	**<0.0001**	**<0.0001**	**0.0419**	0.28	**0.0066**	0.09
Pre-Descemet stromal layer (n=127)									
r_s_	-0.04	**-0.264**	**-0.215**	**-0.485**	**0.409**	0.137	0.14	-0.147	-0.105
q	0.80	**0.0104**	**0.0458**	**<0.0001**	**<0.0001**	0.25	0.25	0.21	0.39

Correlations between various analyzed parameters were calculated using Spearman rank test with FDR correction.

Results with statistical significance are indicated in bold.

### 3.6 Changes of corneal stromal microdots in patients with DR

**Figure 2:**
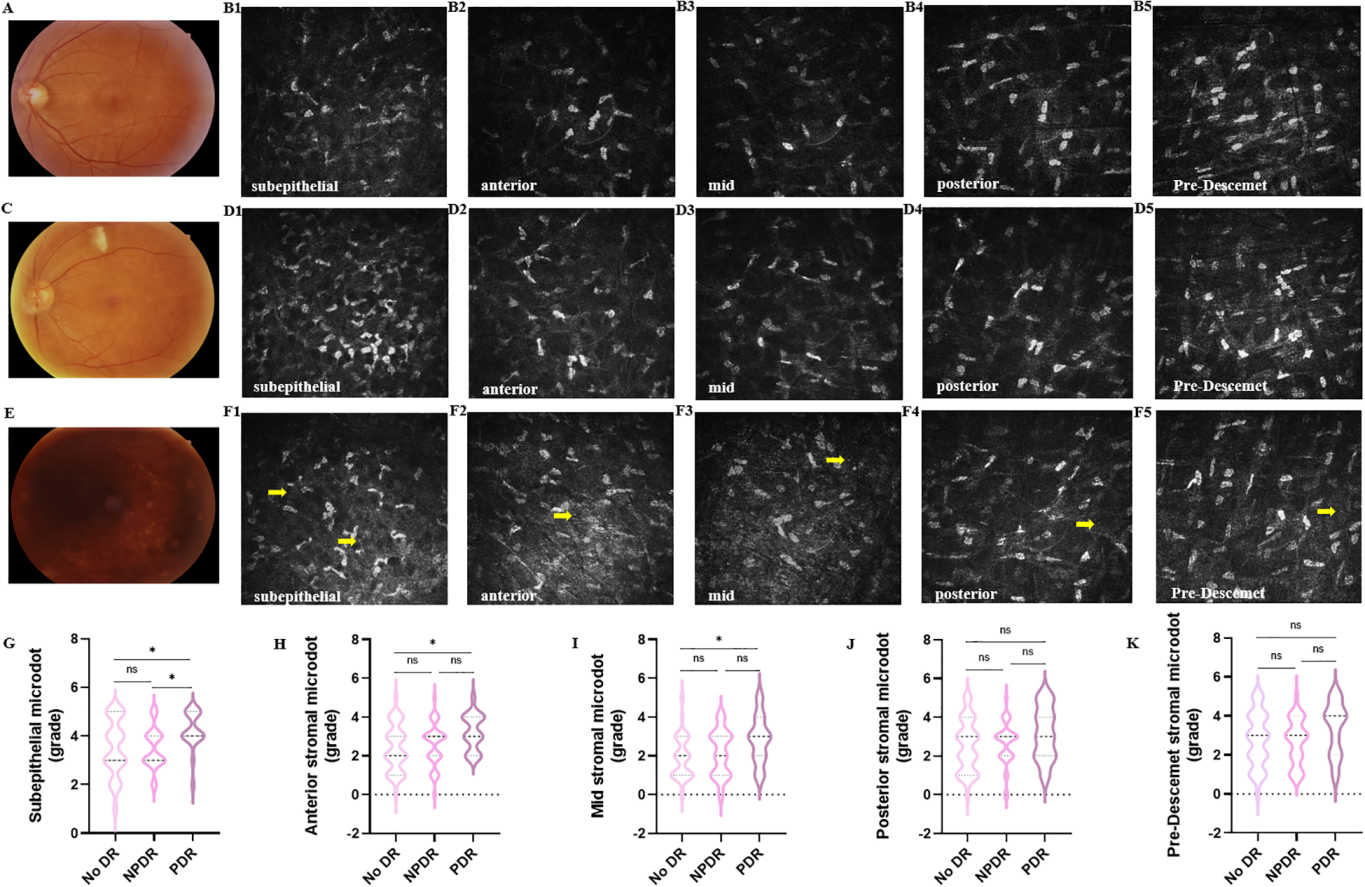
Changes of corneal stromal microdots in patients with DR. Representative color fundus photographs **(A, C, E)** and IVCM **(B1-B5, D1-D5, F1-F5)** images of microdots (yellow arrow) obtained from no DR **(A, B1-B5)**, NPDR **(C, D1-D5)**. and PDR **(E, F1-F5)** eyes. **(G-K)** The comparison of microdots in subepithelial layer **(G)**, anterior stromal layer **(H)**, mid stromal layer **(I)**, posterior stromal layer **(J)**, and pre-Descemet stromal layer **(K)** among no DR, NPDR, and PDR eyes. (*P < 0.05).

Both SVC VD and DVC VD were lower in the DM group than in the healthy control group with 49.10% [Q25–Q75: 46.40–52.10%] vs. 52.75% [Q25–Q75: 50.73–54.50%], *P*<0.0001 and 50.39 ± 7.00% vs. 53.84 ± 4.99%, *P*=0.0014, respectively. There was no significant difference in FAZ between healthy subjects and patients with DM (0.30 ± 0.11 mm^2^ vs. 0.32 ± 0.11 mm^2^, *P*=0.15). Then, we compared the deposition of microdots in no DR, NPDR, and PDR eyes (**Figure 2**). The grade of microdots in the subepithelial layer was higher in PDR eyes than NPDR and no DR eyes (4 [Q25–Q75: 4-5] vs. 3 [Q25–Q75: 3-4], *P*=0.026 and 4 [Q25–Q75: 4-5] vs. 3 [Q25–Q75: 3-5], *P*=0.025). The microdots in anterior and mid stromal layer were higher in PDR eyes than no DR eyes (3 [Q25–Q75: 2-4] vs. 2 [Q25–Q75: 1-3], *P*=0.020 and 3 [Q25–Q75: 2-4] vs. 2 [Q25–Q75: 1-3], *P*=0.012). while the grade of microdots in posterior and pre-Descemet stromal layers had no significant differences among no DR, NPDR, and PDR eyes (posterior, 3 [Q25–Q75: 1-4] vs. 3 [Q25–Q75: 2-3] vs. 3 [Q25–Q75: 2-4], respectively, *P*=0.21; pre-Descemet, 3 [Q25–Q75: 2-4] vs. 3 [Q25–Q75: 2-4] vs. 4 [Q25–Q75: 2-4], respectively, *P*=0.17).

### 3.7 Correlation of microdots with retinal vessel density in patients with DM

The deposition of microdots in subepithelial layer (r_s_=-0.432, *q*<0.0001; r_s_=-0.366, *q*=0.0001), anterior stromal layer (r_s_=-0.320, *q*=0.0011; r_s_=-0.262, *q*=0.0109), and mid stromal layer (r_s_=-0.347, *q*=0.0004; r_s_=-0.293, *q*=0.0037) had negative correlation with SVC VD and DVC VD (**Supplementary Figure 3**). Also, the microdots in the posterior stromal layer had a positive correlation with SVC VD (r_s_=-0.276, *q*=0.0066) and did not correlate with DVC VD (r_s_=-0.187, *P*=0.0354). The microdots in the pre-Descemet stromal layer had no correlation with SVC VD (*P*=0.10) and DVC VD (*P*=0.24) (**Table 3**).

### 3.8 Lipofuscin in DM corneas and healthy corneas

**Figure 3:**
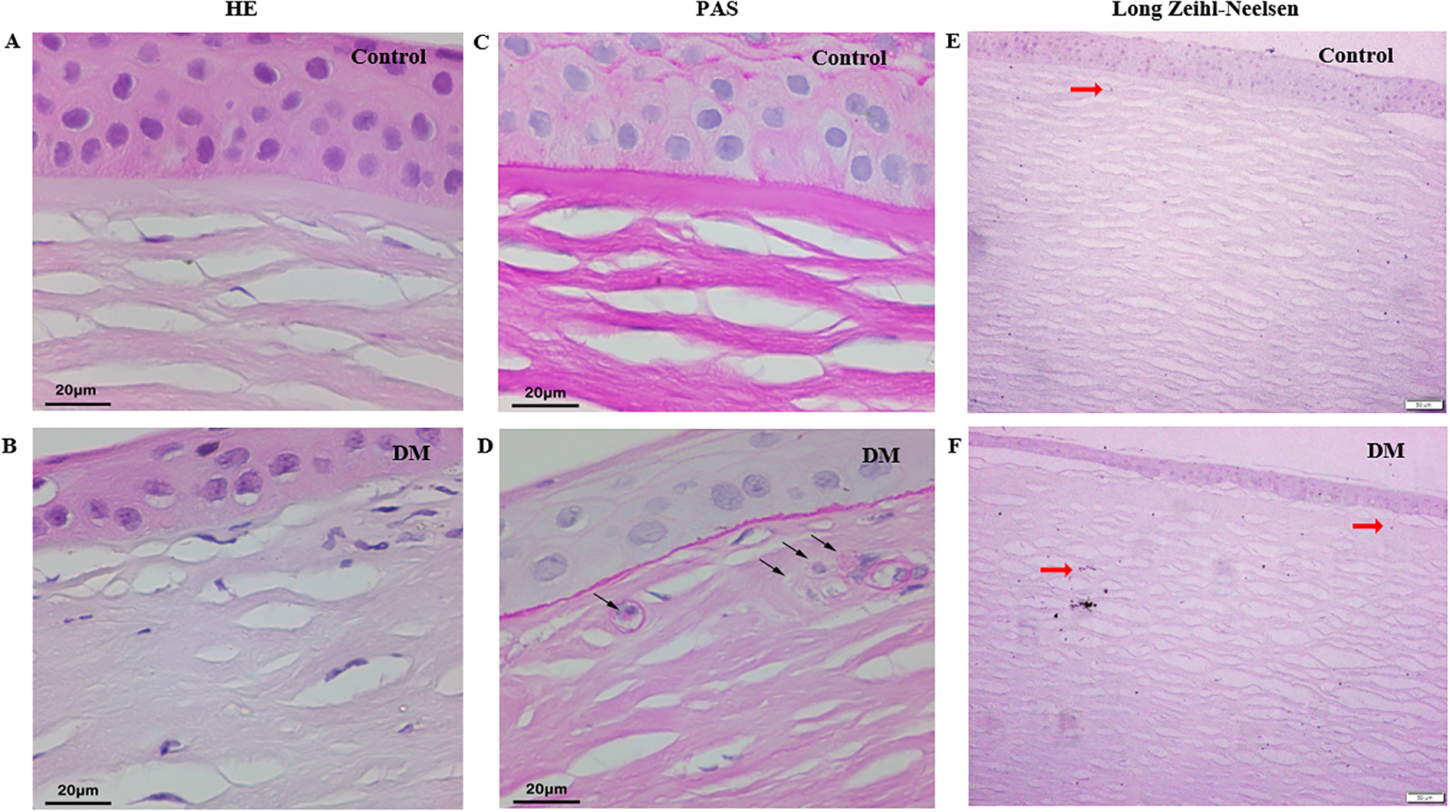
Histochemistry staining of cadaver corneas from DM and control subjects. **(A, B)** HE staining of corneas from control and DM subject (original magnification, X400). **(C, D)** PAS staining of corneas from control and DM subject (black arrow: PAS-positive granules, original magnification, X400). **(E, F)** Long Ziehl-Neelsen staining of corneas from control and DM subject (red arrow: Zeihl-Neelsen-positive granules, original magnification, X100).

HE staining of control and DM corneas showed vacuolated cytoplasm in the DM corneas (**Figures 3A, B**). PAS staining revealed PAS-positive granules in cytoplasm in some keratocytes of DM corneas, whereas control corneas showed minimal staining (**Figures 3C, D**). Additionally, long Ziehl-Neelsen staining exhibited strong positive staining for lipofuscin, manifested as dark brown-black granules in the cytoplasm of some keratocytes in both control and DM corneas, appearing more abundant in the latter (**Figures 3E, F**).

### 3.9 Lipofuscin in diabetic corneal basal epithelial layer

**Figure 4:**
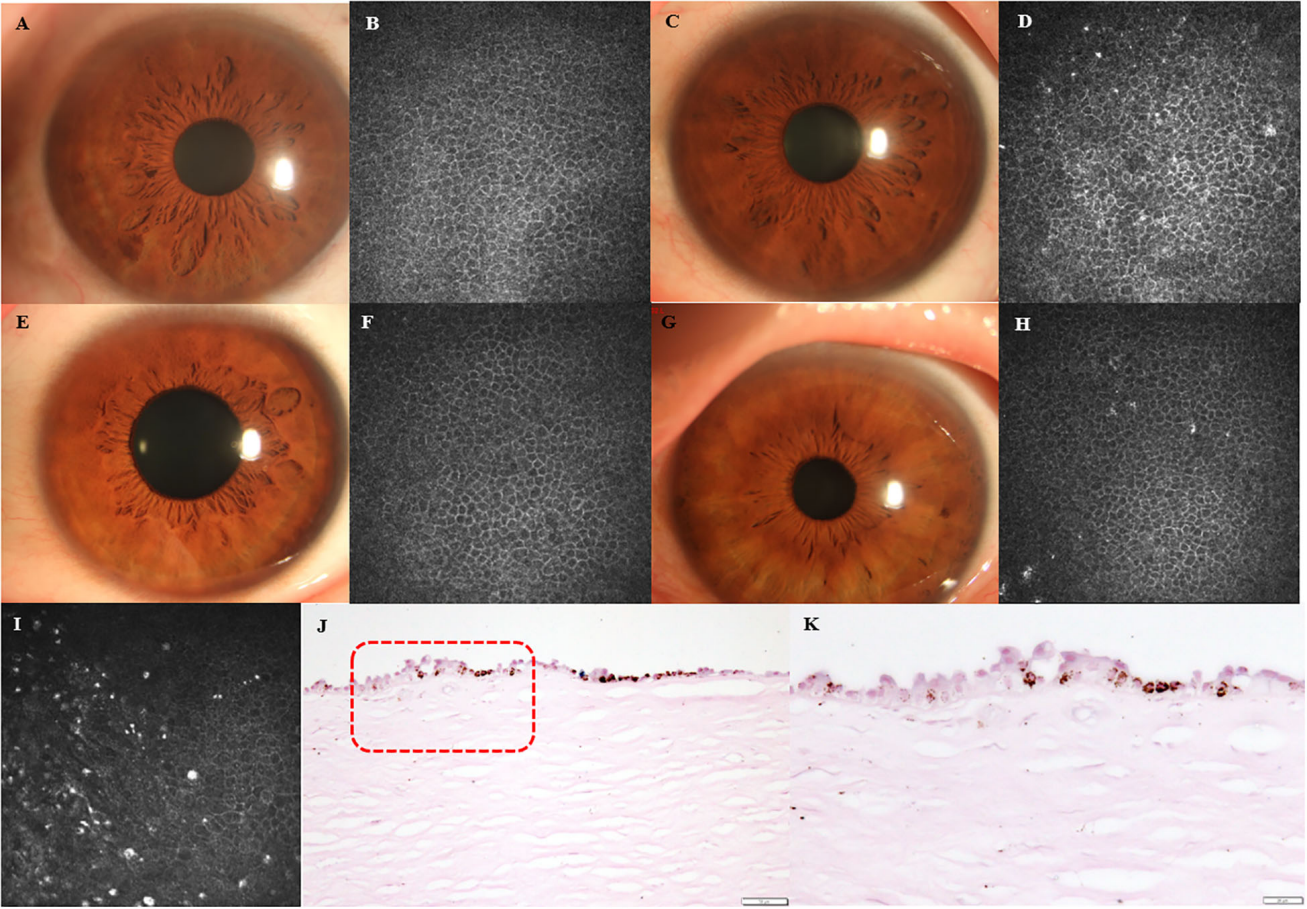
Lipofuscin in corneal basal epithelial layer. **(A, B)** Anterior segment photographs and IVCM images of healthy eye without microdots in corneal basal epithelial layer. **(C, D)** Anterior segment photographs and IVCM images of healthy eye with microdots in corneal basal epithelial layer. **(E, F)** Anterior segment photographs and IVCM images of DM eye without microdots in corneal basal epithelial layer. **(G, H)** Anterior segment photographs and IVCM images of DM eye with microdots in corneal basal epithelial layer. **(I-K)** IVCM image and long Ziehl-Neelsen staining of DM cornea. Higher magnification of the boxed area indicated in **(J)** (original magnification, X200) is presented in **(K)** (original magnification, X400).

Highly reflective round-shape microdots were also found in corneal basal epithelial layer in some of DM eyes (32 in 127 eyes) and healthy eyes (3 in 36 eyes) using IVCM (*P*=0.030) (**Figures 4A-H**). **Figures 4A, B** presented anterior segment photograph and IVCM image of a healthy eye without microdots accumulation in the corneal basal epithelial layer, while **Figures 4C, D** showed those of a healthy eye with microdots. **Figures 4E, F** depicted anterior segment photograph and IVCM image of a DM eye without microdots in the corneal basal epithelial layer, and **Figures 4G, H** illustrated a DM eye with microdots in the corneal basal epithelial layer. Using long Ziehl-Neelsen staining, we found lipofuscin deposition in the basal epithelial layer of one cornea from a patient whose cause of death was dilated cardiomyopathy with 5-year duration of DM (**Figures 4I-K**).

## 4 Discussion

As one of the great healthcare challenges of the 21^st^ century, diabetes has become an increasing threat not only in developed but also in developing countries (1). Diabetes is a group of metabolic diseases characterized by chronic hyperglycemia and is associated with long-term damage to various organs, especially the eyes (2). To our knowledge, this is the first study to assess microdots in patients with DM using IVCM. In this study, we found that the stromal microdots significantly increased in patients with DM. Furthermore, the results also showed that the deposition of microdots in corneal stroma had correlations with corneal nerve loss as well as retinal microvascular perfusion.

Microdots were found as highly reflective spherical structures, about 1-2μm in diameter, and can be observed throughout the entire depth of the corneal stroma (13). Böhnke et al. (5) first found these microdots deposited in patients with a long-term contact lens history, especially in the soft contact lens group, while they found no microdot deposits in healthy people. Soon after, Trittibach et al. (13) observed the same situation that microdots only deposited in contact lens wearers but didn’t deposit in healthy volunteers. However, Efron et al. (16), found that microdots can be observed in virtually all corneas from all age groups and appear to be more prevalent in older subjects. Also, Hillenaar et al. (16) and Utheim et al. (11) observed microdots in healthy volunteers, and they found a correlation between age and the grade of microdots. In the present study, we observed microdots in most of the eyes of examined healthy people and patients with DM who were age-matched and had no history of contact lens wear.

Microdots have been reported in many diseases, including Thygeson’s superficial punctate keratopathy (6), amiodarone-induced keratopathy (9, 17, 18), Fabry disease (8, 9, 17), and mustard gas keratopathy (19). While, whether there was a change in microdots in DM has not previously been investigated. At present, we observed an increase in the grade of microdots throughout the entire corneal stroma. Considering the former study which suggested that these microdots form as a result of chronic oxygen deprivation and chronic inflammatory stimulus to the cornea (5), we assumed that the change of microdots correlates with the duration of DM. Our result proved that the microdots in the subepithelial layer correlate with the duration of DM. However, microdots in other layers didn’t correlate with the duration of DM.

Previous research already provides evidence for early corneal neuronal loss even in patients with DM with short mean durations of the disease (20). Numerous studies have revealed changes in corneal cell density and nerves in patients with DM (21, 22). In consistent with precious research, we also found a decrease in corneal basal epithelial density, endothelial density, CNFD, CNBD, and CNFL. Corneal nerve fiber alterations have been considered to progress in parallel with the disease (23). In our study, we found that the deposition of microdots had a positive correlation with corneal nerve fiber alterations, suggesting that stromal microdots might be used to evaluate the severity of DM. The alteration in DC density in diabetic corneas was in debate. The majority of research found that dendritic cells accumulated in DM corneas (24, 25), while Gao et al. (26) found out that corneal DC density reduced in streptozotocin-induced type 1 diabetic mice. In our study, corneal DC density was increased in diabetic corneas, suggesting a low-grade inflammation in DM. Also, we found a positive correlation between stromal microdots and DC density except in the pre-Descemet stromal layer, which suggests that the deposition of microdot might correlate with corneal inflammation, while further investigation is required to determine whether inflammation precedes or follows the deposition of microdots. The differences among various severity of DR were also compared in the study. We found that microdots in the subepithelial, anterior stromal, and mid stromal layer increased in PDR eyes when compared with no DR eyes. Although the grade of microdots in other layers had no significant differences among no DR, NPDR, and PDR eyes, the deposition of microdots showed a slight increase in PDR eyes. We thought that the reason for this result is the insufficient sample size of the NPDR (n=33) and PDR (n=19) patients. In the future, we will expand the sample size for further observation.

Previous research already provided evidence for early corneal neuronal loss and retinal microvascular changes in patients with diabetes with short mean durations of the disease and without clinically visible signs of DR (20, 27, 28). In this study, we found the stromal microdots had correlations with both corneal neuronal loss and retinal microvascular perfusion, which indicates that microdots might deposit from the early stage of DM. The negative correlations between microdots and CNFD or CNFL, along with their positive associations with nerve tortuosity and dendritic cell density, suggest that microdots may not merely result from aging or lipid accumulation but may actively participate in or reflect corneal neurodegenerative processes. Furthermore, their association with reduced vessel density in the superficial and deep vascular complexes (SVC and DVC) implies that microdots could serve as indirect biomarkers of retinal microvascular dysfunction. Further mechanistic studies are needed to clarify the role of microdots in diabetic neurovascular pathology.

The origin and structural/functional significance of microdots is barely known. Böhnke et al. (5) thought that microdots may be lipofuscin or some other highly reflective matter well maintained in tissues. Efron et al. (29) gave the thought that microdots may represent dysgenic or apoptotic cellular remnants. Lipofuscin is a complex of oxidized proteins (30-70%) and lipids (20-50%) thought to be derived from the peroxidation of polyunsaturated lipids of subcellular membranes and is characterized in tissue sections by being positive to periodic acid-Schiff (PAS) staining, acid fast by the long Ziehl-Neelson technique and autofluorescence (30). Hidayat et al. (31) observed lipofuscin, 1-3μm in size, in patients with chronic keratitis and corneal bilateral opacities. In this study, we found lipofuscin granules accumulated in corneal stroma by PAS staining and long Ziehl-Neelson technique both in patients with DM and in healthy volunteers. In this study, we also found a correlation between microdots and LDL, TC, as well as ApoB. These results indicated that microdots are at least partially composed of lipofuscin. Also, we found lipofuscin deposited in the DM corneal basal epithelial layer.

Our study has some limitations. First of all is the limited cornea samples. In future studies, we will make more observations in corneal basal epithelial layer of DM corneas using IVCM and histochemistry staining. In addition, although statistical significances were detected in correlative analysis, most of which were weak to moderate correlations (r_s_<0.5). Studies with larger sample sizes are needed to verify the results in the future.

## Data Availability

The original contributions presented in the study are included in the article/**Supplementary Material**. Further inquiries can be directed to the corresponding author/s.
