# Supplementary material for: Corneal stromal microdots accumulation and its association with corneal neurodegeneration and retinal microvascular perfusion in diabetes
Source: Front Endocrinol (Lausanne). 2025 Aug 27;16:1481018. doi: 10.3389/fendo.2025.1481018 (PMC12421287; doi:10.3389/fendo.2025.1481018)
Supplement: Supplementary file 1 [file DataSheet1.docx]

Supplementary Table 1**.** Comparison of corneal nerve morphology and corneal cell density in DM patients and control group.

|  | **Control subjects**  **(n = 36)** | **Diabetes**  **(n =127)** | **T/Z** | ***P* Value** |
| --- | --- | --- | --- | --- |
| Basal epithelial cell density  (cells/mm^2^) | 6144.5 (5853.5, 6417.5) | 5876 (5213, 6363) | -2.31 | **0.0210** |
| CNFD (number/mm^2^) | 32 (26, 39) | 17 (13, 25) | -6.97 | **<0.0001** |
| CNBD (number/mm^2^) | 80 (59.5, 99.75) | 57 (44, 71) | -4.04 | **<0.0001** |
| CNFL (mm/mm^2^) | 18.65 ± 3.55 | 11.38 ± 4.38 | 9.14 | **<0.0001** |
| Tortuosity (grade) | 1 (1, 1) | 2 (2, 3) | -6.38 | **<0.0001** |
| DC density (cells/mm^2^) | 4 (0, 13) | 13 (6, 27) | -4.06 | **<0.0001** |
| Endothelial cell density  (cells/mm^2^) | 3104 (2726.75, 3295) | 2778 (2479, 3024) | -3.58 | **0.0003** |

Data are shown as mean ± standard deviation or median (Q25-Q75).


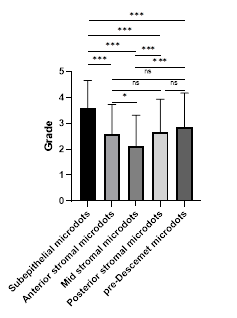


**Supplementary Fig. 1.** Comparison of corneal stromal microdots among five groups revealed that the subepithelial layer had the highest deposition of microdots.


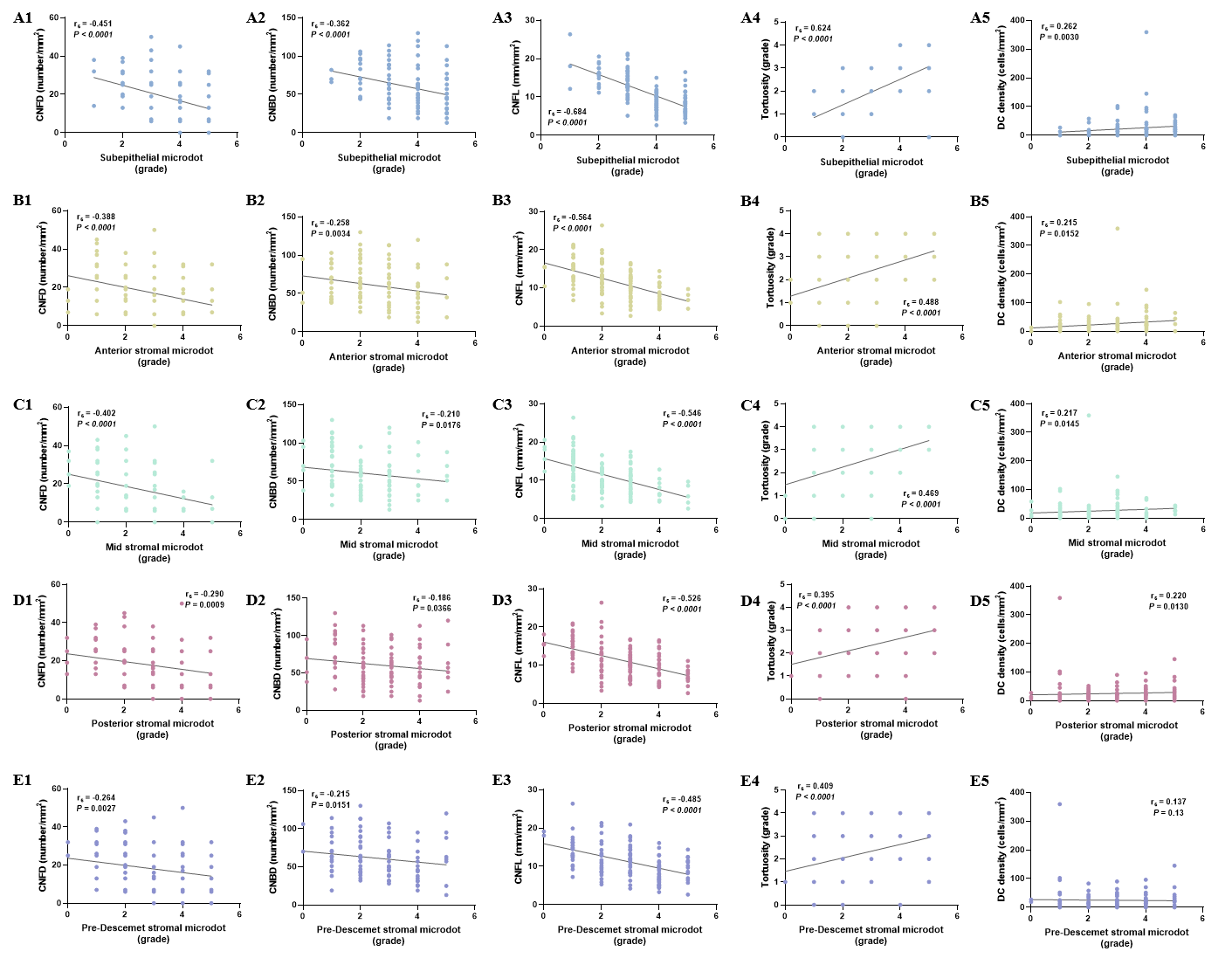


**Supplementary Fig. 2. Correlation of Microdots with Corneal Nerve and Corneal Cell Density in DM Patients.** Spearman’s rank correlation coefficient (r_s_) and *P* values were shown in the figure. *P* value of less than 0.05 was considered statistically significant.


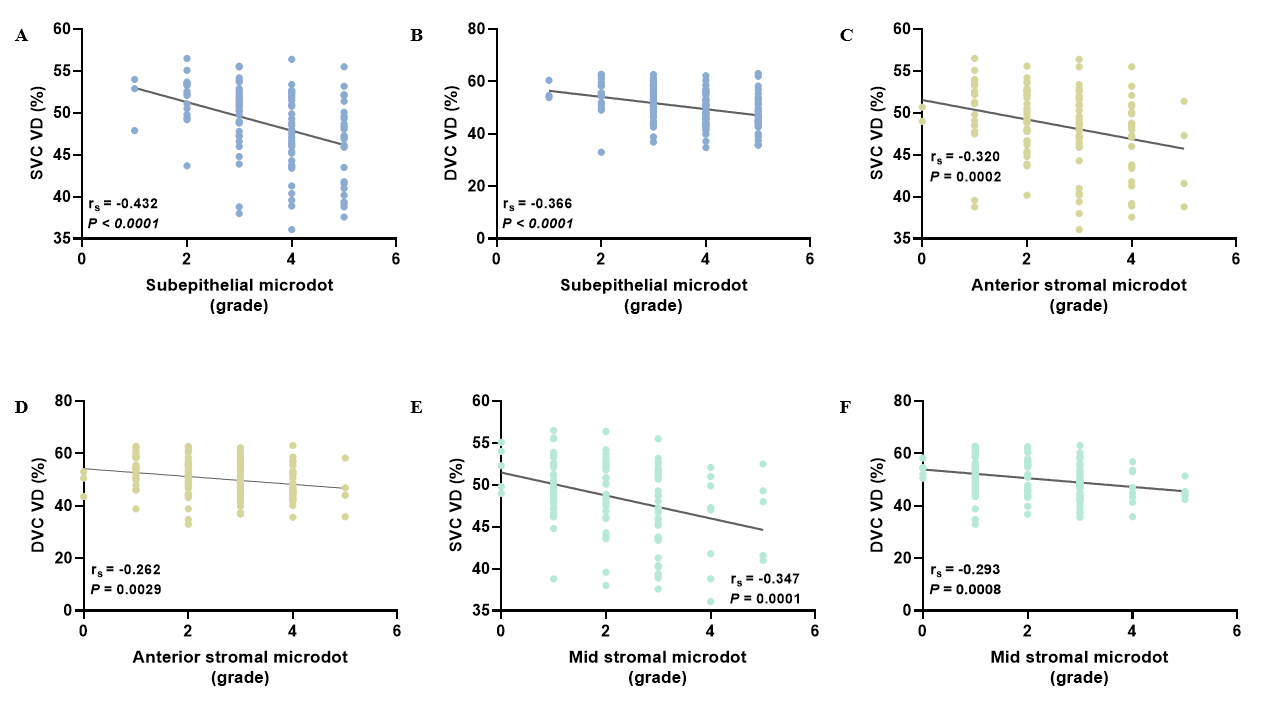


**Supplementary Fig. 3. Correlation of Microdots with Retinal Vessel Density in DM Patients.** Spearman’s rank correlation coefficient (r_s_) and *P* values were shown in the figure. *P* value of less than 0.05 was considered statistically significant.
